# A remarkable new *Awas* Löbl from southern China (Coleoptera, Staphylinidae, Pselaphinae)

**DOI:** 10.3897/zookeys.522.6109

**Published:** 2015-09-23

**Authors:** Zi-Wei Yin, Jia-Wei Shen, Li-Zhen Li

**Affiliations:** 1 Department of Biology, College of Life and Environmental Sciences, Shanghai Normal University, 100 Guilin Road, Shanghai, 200234, P. R. China

**Keywords:** *Awas*, new species, myrmecophile, *Pachycondyla*, Oriental region

## Abstract

A new distinctive species of the rare Oriental goniacerine genus *Awas* Löbl, *Awas gigas* **sp. n.**, is described and illustrated, based on three males and fourteen females taken at the Daoyao Shan Natural Reserve in the southern Chinese province of Guangxi. All specimens were collected from colonies of the ant genus *Pachycondyla* F. Smith nesting in decomposing woods.

## Introduction

The genus *Awas* Löbl currently contains six described species scattered throughout the Malay Peninsula, Taiwan, and continental China (Löbl 1994; Nomura 1995; Nomura and Idris 2004; Yin, Li and Zhao 2010; Yin and Li 2012). After a discussion of the morphological details and a phylogenetic analysis of the genus, Löbl (1994) placed *Awas* in the goniacerine tribe Arnylliini, as a sister taxon of *Harmophorus* Motschulsky. Members of *Awas* are unique in having a conspicuously elongate postocular region of the head, and a relatively small, basally strongly constricted abdomen in contrast to the large body.

All *Awas* species are rare in scientific collections, known from one (*Awas giraffa* Löbl, *Awas sinicus* Yin & Li, *Awas kayan* Yin & Li, *Awas loebli* Yin & Li), four (*Awas rajah* Nomura & Idris), and five (*Awas shunichii* Nomura) specimens (additional records for *Awas rajah* and *Awas shunichii* provided in Sugaya and Nomura 2003; Nomura and Idris 2005). Information of the habitat of the known species are largely limited due to the inadequate number of specimens: *Awas giraffa*, *Awas shunichii*, *Awas sinicus*, and *Awas kayan* were collected from leaf litter, and *Awas rajah* and *Awas loebli* were taken by flight intercept traps.

In July 2014, our team collected two males and two females of an additional species from a colony of a *Pachycondyla* ant at the Dayao Shan Natural Reserve in the southern Chinese province of Guangxi. With the knowledge of the host ant, a second survey in the same locality was conducted in May 2015, and another thirteen specimens (one male, twelve females) were found in several colonies of the same ant species. Based on the above material, a new species is formally described, and compared to the known congeners. This species is distinct in having the largest body size of more than 5.0 mm.

## Material and methods

All material treated in this paper is housed in the Insect Collection of Shanghai Normal University (SNUC), Shanghai, China.

A slash is used to separate different labels. Authors’ notes are included in brackets. Each type specimen bears a following label: ‘HOLOTYPE (red), or PARATYPE (yellow), ♂ (or ♀), *Awas gigas* sp. n., det. Zi-Wei Yin, 2015’.

The following abbreviations are applied: AL – length of the abdomen along the midline; AnL – length of the antenna; AW – maximum width of the abdomen; EL – length of the elytra along the sutural line; EW – maximum width of the elytra; HL – length of the head from the anterior clypeal margin to the occipital constriction; HW – width of the head across eyes; PL – length of the pronotum along the midline; PW – maximum width of the pronotum. Length of the body is a combination of HL, PL, EL, and AL.

## Description

### Awas gigas sp. n.

Taxon classificationAnimaliaColeopteraStaphylinidae

http://zoobank.org/87EE660C-C902-44AF-AA1C-DA3B20A4A817

Figs 1 , 2 , 3

#### Type material

(3 ♂♂, 14 ♀♀)**. Holotype** (in SNUC): **CHINA**: ♂, labeled ‘China: Guangxi, Jinxiu Hsien (金秀县), Dayao Shan N. R. (大瑶山自然保护区), 16 km (16公里), 24°08'11"N, 110°14'28"E, *Fagus* forest, rotten woods, colony of *Pachycondyla* ant, 1100 m, 17.vii.2014, leg. Z. Peng’. **Paratypes** (in SNUC): **CHINA**: 1 ♂, 2 ♀♀, same label data as the holotype; 1 ♀, same locality, ‘16 km, 24°08'25"N, 110°15'38"E, 960 m, colony of *Pachycondyla* ants, 01.vi.2015, leg. J.-W. Shen & Z. Peng’; 1 ♂ (antennomeres VII–VIII closely conjoint, deformed status), 11 ♀♀, also Dayao Shan N. R., ‘Laoshan Station (老山林场), 24°07'02"N, 110°11'51"E, 950 m, *Pachycondyla* ant, 31.v.2015, leg. J.-W. Shen & Z. Peng’.

#### Diagnosis.

Body large-sized, length 4.79–5.12 mm; head with a greatly elongate postocular region; pronotum relatively stout, basolateral margins moderately incised at level of antebasal sulcus, lacking distinct setal tufts; elytra lacking basal fovea. Female has a relatively larger abdomen than male.

#### Description.

**Figure 1.**
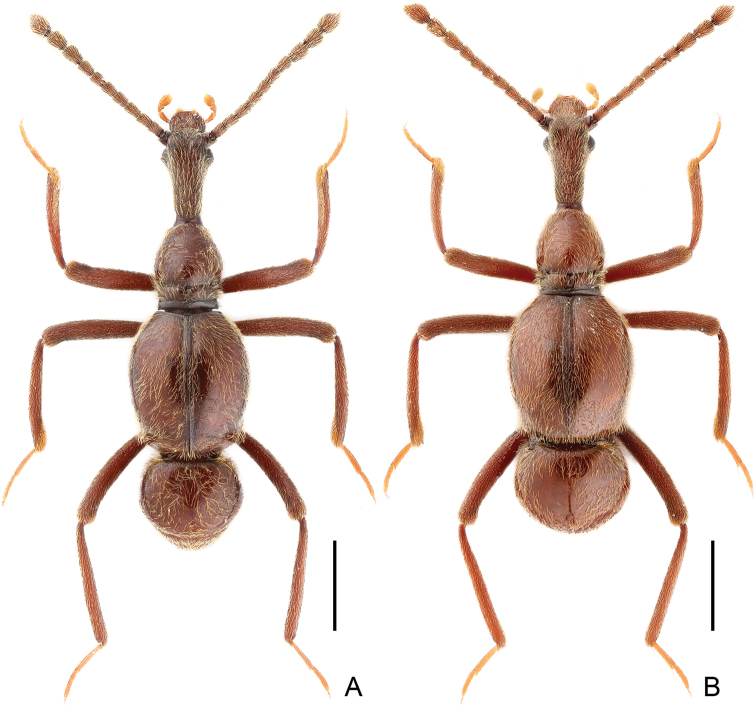
Dorsal habitus of *Awas gigas*. **A** Male **B** Female Scales: 1.0 mm.

**Figure 2.**
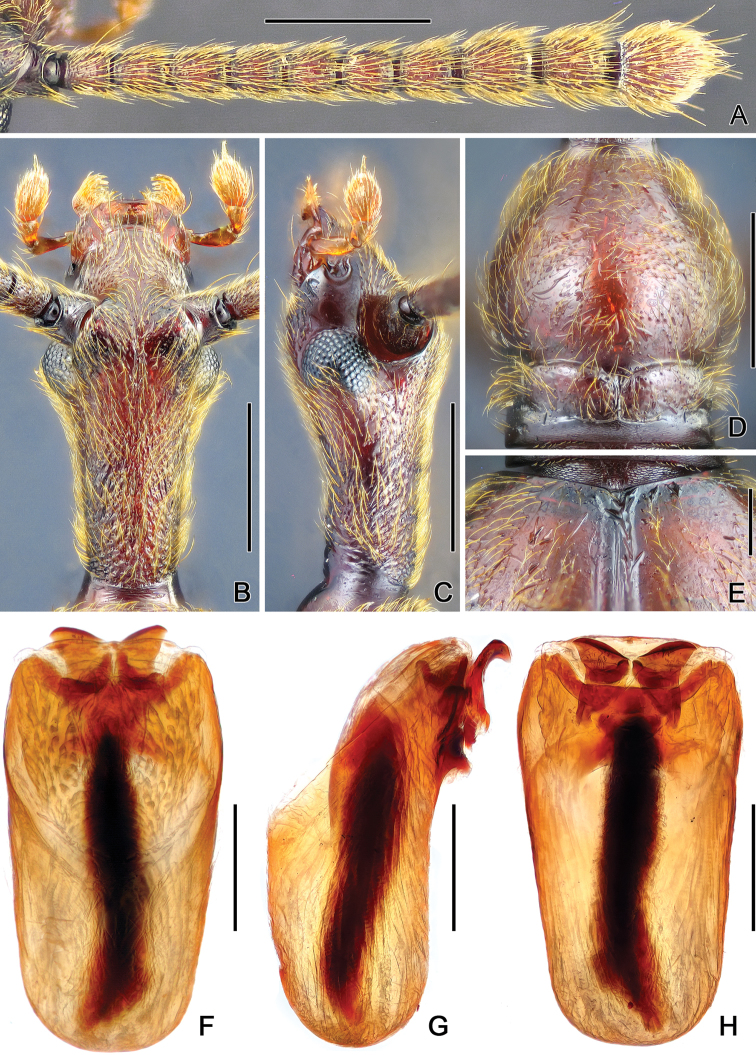
Diagnostic characters of male *Awas gigas*. **A** Right antenna **B** Head, in dorsal view **C** Same, in lateral view **D** Pronotum **E** Elytral base **F** Aedeagus, in dorsal view **G** Same, in lateral view **H** Same, in ventral view. Scales: A–D = 0.5 mm, E–H = 0.2 mm.

Male (Fig. 1A). BL 4.79–4.96 mm; body reddish-brown, mouth parts and tarsi lighter. Head (Fig. 2B–C) strongly elongate, HL 1.24–1.35 mm, HW 0.59–0.61 mm, densely punctate and roughly sculptured; pubescence directed anteriorly; anterior frontal margin roundly protruding medially; postocular margins gradually narrowed toward occipital constriction; gula slightly depressed, foveae in longitudinal slit; eyes prominent, situated anterior head mid-length, each eye composed of about 95 facets; maxillary palpi with palpomeres I short, II elongate, slightly expanded apically, III nearly triangular, IV oval, with long, membranous apical palpal cone; AnL 2.13 mm, antennomeres IX–XI (Fig. 2A) wider than previous ones. Pronotum (Fig. 2D) longer than wide, PL 0.91–0.94 mm, PW 0.74–0.78 mm; finely punctate, with T-shaped antebasal sulcus; posterior margin with band of transverse microsculpture. Prosternum with dense admesal pubescence, pubescence on lateral margins sparser. Elytra slightly longer than wide, EL 1.50–1.54 mm, EW 1.35–1.41 mm, widest at basal two-fifths, rounded laterally, narrowed basally and apically, lacking basal fovea (Fig. 2E), with complete sutural striae, densely setose. Legs slender, profemora with indistinct preapical denticle. Abdomen about as long as wide, AL 0.96–1.31 mm, AW 1.18–1.20 mm; tergite IV largest, basolateral margins densely setose. Aedeagus (Fig. 2F–H) symmetric, length 0.66–0.67 mm; median lobe truncate apically; endophallus with hair-like structure; with ventrally curved hook-like parameres.

Female (Fig. 1B). Similar to male in general, size larger; with relatively larger abdomen; each eye composed of about 75 facets. Measurements: BL 4.89–5.12 mm, HL 1.26–1.28 mm, HW 0.56–0.57 mm, AnL 2.03–2.13 mm, PL 0.92–0.93 mm, PW 0.76–0.77 mm, EL 1.54–1.61 mm, EW 1.37–1.39 mm, AL 1.17–1.30 mm, AW 1.27–1.28 mm.

#### Comparative notes.

At first glance *Awas gigas* is very distinct from other species in the genus by possessing a large body size. It shares with *Awas giraffa* and *Awas rajah* the lack of two pairs of setose tufts on the basolateral margins of the pronotum, and lack of a distinct basal elytral fovea, but can be separated from both by the relatively stouter pronotum. *Awas kayan* also lacks distinct pronotal setose tufts, but has each elytron possessing a well-defined basal fovea, and the elytra are broader at basal third.

#### Biology.

**Figure 3.**
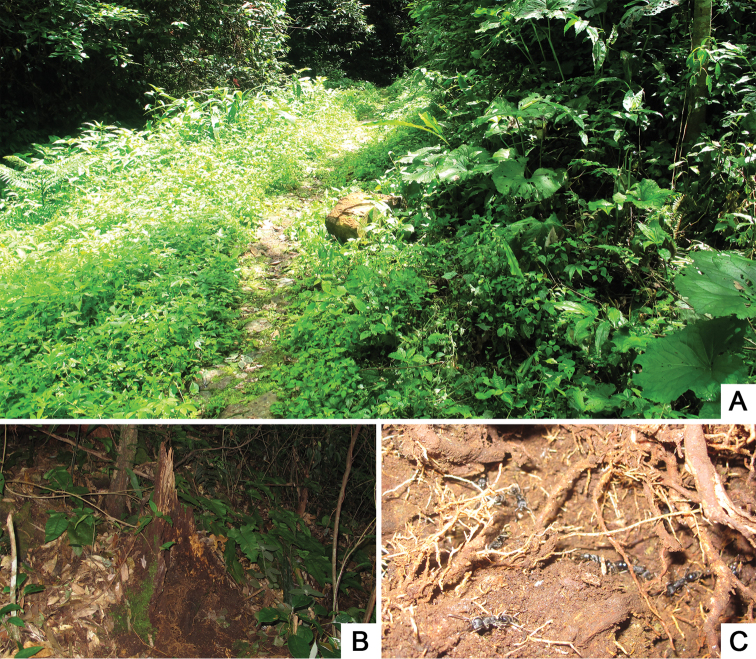
Habitat of *Awas gigas*. **A** General environment of the collection site **B** A decomposing wood from where a colony of *Pachycondyla* was found **C** Inside of the ant colony.

All individuals of *Awas gigas* were collected from colonies of a *Pachycondyla* ant nesting inside or under decomposing woods in broad-leaved forests (Fig. 3).

#### Distribution.

Southern China: Guangxi.

#### Etymology.

The specific epithet indicates the large body size of the new species.

## Supplementary Material

XML Treatment for Awas gigas
